# Needs, Priorities, and Recommendations for Engaging Underrepresented Populations in Clinical Research: A Community Perspective

**DOI:** 10.1007/s10900-016-0279-2

**Published:** 2016-11-03

**Authors:** Jennifer Cunningham Erves, Tilicia L. Mayo-Gamble, Alecia Malin-Fair, Alaina Boyer, Yvonne Joosten, Yolanda C. Vaughn, Lisa Sherden, Patrick Luther, Stephania Miller, Consuelo H. Wilkins

**Affiliations:** 1 Department of Internal Medicine, Meharry Medical College, Nashville, TN 37208 USA; 2 Department of Family and Community Medicine, Meharry Medical College, Nashville, TN 37208 USA; 3 Vanderbilt Institute for Clinical Translational Research (VICTR), Vanderbilt University Medical Center, Nashville, TN 37203 USA; 4 National Health Care for the Homeless Council, Nashville, TN USA; 5 Vanderbilt University School of Medicine and Vanderbilt Institute for Medicine and Public Health, Nashville, TN 37203 USA; 6 Meharry-Vanderbilt Alliance and Vanderbilt University, Nashville, TN 37208 USA; 7 Johns Hopkins Center to Reduce Cancer Disparities, Baltimore, MD 21287 USA; 8 Nashville CARES, Nashville, TN 37204 USA; 9 Department of Surgery, Meharry Medical College, Nashville, TN 37208 USA; 10 Meharry-Vanderbilt Alliance, Vanderbilt University Medical Center, Meharry Medical College, 1005 Dr. D.B. Todd Jr. Blvd., BioMedical Building, Nashville, TN 37208 USA

**Keywords:** Underrepresented populations, Research studies, Community engagement, Health and healthcare disparities

## Abstract

Engaging underrepresented groups in outcomes research is a public health priority for reducing health and health care disparities; yet, engaging these groups is challenging. Failure to involve these underrepresented populations in research further exacerbates these disparities. This article presents the health and research priorities of diverse groups of underrepresented populations in biomedical research, their concerns for participating in research, and strategies to engage them in their healthcare and research studies. Eleven community listening sessions, ranging from 7 to 13 community members each (N = 117), representing racial/ethnic minority, economically disadvantaged (e.g., uninsured), and hearing impaired communities. We used an inductive, qualitative content analysis approach to analyze the data for emerging themes. We identified the following themes: Uncertainties of underrepresented populations regarding research participation; Ineffective communication about research opportunities and research findings; Research on primary care and prevention are priorities for underrepresented populations in research; and Research teams need training in cultural competence and humility. Underrepresented groups provided research priorities, concerns, and strategies to engage them in their healthcare and in research studies. Findings from this study could facilitate improvement of research participation among underrepresented groups, ultimately reducing health disparities and improving quality of life among groups commonly omitted from research recruitment and participation.

## Introduction

Community participation in health research serves as a catalyst for the development of best practices, systemic changes in healthcare delivery, and improved health outcomes [2, 28, 32]. Despite major advances in medicine and public health due to research [25], diverse representation in research participation remains a challenge. Researchers struggle to access, engage, recruit, and retain underrepresented populations such as racial and ethnic minorities, those from socio-economically disadvantaged backgrounds, and/or individuals with disabilities [3, 6, 15, 29]. From the research perspective, this limits understanding of the effectiveness of a given treatment across populations, discovery of new drugs and devices, improved healthcare delivery, and knowledge of behavioral and physical science [1, 3]. From the patient perspective, there is limited understanding of underrepresented population needs, and could be a major component of the observed health and healthcare disparities. Studies have suggested that their absence in research exacerbates their vulnerability to poor health outcomes and reduced access to healthcare services [1, 3]. Hence, increasing diversity in research via engaging underrepresented populations is important for reducing the unequal burden of disparities in health and healthcare [4].

There is a growing awareness by researchers of the critical need to engage underrepresented populations in research [12], resulting in studies to explore this phenomenon. Past findings suggest that underrepresented individuals are willing to participate in research [14, 23, 24], and have expressed positive views about research participation being an opportunity or even a right [23]. However, barriers reduce researchers’ ability to engage with these groups. These individuals are often not in an environment where research recruitment occurs [20], and/or they lack awareness of research opportunities. Underrepresented groups sometimes feel that research is not relevant to them or their families [14]. Limited knowledge about the nature of research is another barrier. For example, these community members often misunderstand the purpose of research and research procedures; perceive that clinical trials will not benefit them personally; feel frustrated due to poor dissemination of research findings to the community; and feel “left out” of the research process [23]. As a result, a new approach is warranted to enhance research participation rates.

Community engagement has been identified as an effective, evidence-based mechanism to improve interest and trust in research [8]. Community engaged research (CEnR) principles such as community member involvement in the research process, seeking input from community advisory boards, and peer-concordant, community health worker led study recruitment have shown promise to enhance clinical trial recruitment and retention [10, 13, 19, 22]. However, opportunities for underrepresented populations to engage in the entirety of the research process (i.e., involvement in early phases such as setting research agendas, or in advisory capacities) are limited. The goal of this study was to use community engaged research principles to identify research priorities and concerns of underrepresented populations. We believe our findings can be used to inform clinical research areas and study design to increase participation of underrepresented populations in health research which may, in turn, advance the understanding of health disparity origins.

## Methods

### Community-Academic Partnership

One of the goals of the Meharry-Vanderbilt Community Engaged Research Core (CERC) is to build academic and community capacity to conduct patient and community engaged research. In order to promote this goal, a collaborative partnership between CERC academic partners, including the Vanderbilt Institute for Clinical and Translational Research (VICTR) and the Meharry Clinical and Translational Research Center (MeTRC), and a community partner, the Neighborhoods Resource Center (NRC) was formed. VICTR and MeTRC were developed with Clinical and Translational Science awards to Vanderbilt University Medical Center from NCATS and to Meharry from NIMHD, respectively. NRC is a community-based non-profit organization that works with residents and neighborhood associations to build stronger neighborhoods, particularly in low-income and marginalized communities. For this study, the NRC was engaged in all phases of this research, from conceptualization to dissemination. Members of the academic-community partnership engaged in several meetings prior to study implementation to plan study logistics. All study procedures were reviewed and approved by the Vanderbilt University Medical Center Institutional Review Board.

### Study Participants

Underrepresented research populations residing in Nashville/Davidson County were sought as participants in this study, where individuals were considered underrepresented if they belonged to a group that does not typically participate in research due to cultural or socioeconomic barriers, or issues related to a physical/cognitive impairment [20]. These populations were chosen based on a review of the literature that demonstrated their lack of participation in research [3, 6, 15, 29].

Participants were sought in specific Nashville urban core neighborhoods densely populated with underrepresented populations where NRC has community relationships and through NRC partner community organizations whose members are those likely to be underrepresented in research. For the data collection, 10 groups of hard-to-reach community members were convened, including low socioeconomic status African American and Spanish/English-speaking individuals, Spanish speaking individuals from a faith-based organization, individuals from economically disadvantaged (i.e., uninsured, HUD housing) backgrounds, and individuals with hearing impairments. Individuals with hearing impairments were not included in the original target population. We added this group of community members when we became aware of this population at CERC’s community research day, an interactive networking session between potential academic and research partners.

### Study Design

A key component of CEnR is engaging community members in prioritizing research topics [8]. The academic-community partnership identified Community Listening Sessions (CLS) as the most appropriate data collection method for this study. Eleven CLSs with a total of 117 individuals were conducted in urban core neighborhoods highly populated with underrepresented populations. A CLS is a qualitative research method where a group of people of diverse backgrounds and perspectives express their views on an important issue. These sessions have shown success because they are led by a well-known, trained community leader and are less structured than focus groups, allowing individuals to express their views on an issue in a more wide-ranging manner [30]; focus groups are led by trained facilitators and involve a focused discussion topic [11, 30].

### Sampling and Recruitment

A purposive sampling method was used to recruit participants. Each participant was screened for inclusion criteria (i.e., adults, targeted neighborhood resident or priority health issues, ethnicity, and ages 18 and over). All eligible participants resided in the targeted neighborhood except for deaf and hard of hearing participants, the Latino participants, and the African-American male participants. Recruitment for the deaf and hard of hearing group was conducted through *Bridges*, an organization that serves deaf and hard of hearing adults and children to ensure they reach their potential [7]. Recruitment strategies included flyers as well as scripted phone and e-mail correspondence to NRC networks (e.g., church and neighborhood associations) to identify potential participants. The Latino participants were recruited using a Spanish-translated flyer, which was distributed in high density areas.

### Study Procedures

#### Community Listening Session Facilitator Training

A facilitator’s guide, developed jointly by CERC and NRC, focused on the following topics: priority health concerns for research, perceptions towards research, methods or sources for receiving health information and barriers to research. Questions included: (1) What are the most important health concerns for you and your family? (2) What health concerns would you like to see being researched or addressed by the health systems? (3) How do you generally receive health information? (4) What is the most effective way for you, your family and community to receive health information? (5) Have you ever participated in research? If yes, was it a positive or negative experience? If you are comfortable, please elaborate. If no, have you ever been asked? Would you participate if asked? (6) In general, do you think research is important to improving health? (7) What concerns do you have about participating in research? (8) What are some of the barriers to participating in research?

Three NRC organizers were trained by CERC staff in reflective techniques that encouraged discussion and enhanced each participant’s sense of having been heard [5]. All facilitators attended a 1.5 h training offered by *Bridges* to learn how to work with an interpreter and basic cultural competencies when working with the deaf and hard of hearing community. For the Latino CLS, a bicultural, bilingual interpreter/translator was recruited to assist in facilitating the session.

#### Community Listening Session Procedures

Nine community listening sessions were held in a community setting such as a familiar neighborhood community center or family resource center. The deaf and hard of hearing listening session was held at *Bridges* and the Latino listening session was held at a Hispanic church. Written informed consent was obtained from all participants. Community NRC organizers and CERC staff facilitated all sessions. Specifically, a bilingual trained facilitator and a CERC staff member conducted the Latino CLS in Spanish. The CLS for the hearing impaired group was conducted by a *Bridges* staff member and a CERC staff member using American Sign Language (ASL). The session was conducted by two interpreters, a trained facilitator, and CERC staff. Community Listening Sessions lasted approximately 1.5 h each. The sessions were audio-recorded and notes were scribed on a flip chart allowing participants to ensure an accurate representation of their comments. All sessions were commercially transcribed and used verbatim for analysis.

### Data Analysis

An inductive, qualitative content analysis approach was used to identify emerging themes [18]. This is a bottom-up approach that inductively builds upon the data as opposed to establishing a deductive coding scheme in advance of the analysis. Two researchers performed coding consensus using a line-by-line open coding technique [16]. Key concepts, ideas, beliefs, and/or events were coded with one or more codes. Codes were added or modified as necessary as new meanings emerged [27]. To assess coding consistency, codes and their assignment to text were checked and rechecked. If any discrepancies arose, researchers discussed the codes until consensus was reached. Using a constant comparison method [16], codes were compared and queried to identify themes that emerged from the groups.

## Results

Eleven listening sessions were conducted with 117 participants from urban core neighborhoods highly populated with underrepresented populations. While the majority of the participants were African Americans (66 %), the sample was racially and ethnically diverse with Caucasians (15 %) and Hispanics (14 %). Seventy-one percent of participants were female. Many (41 %) of the participants were over the age of 50, followed by the age ranges of <30 (21 %), 30–39 (19 %), and 40–49 (16 %).

Four themes emerged from community member responses: (1) Uncertainties of underrepresented populations regarding research participation; (2) Ineffective communication about research opportunities and research findings; (3) High priorities for underrepresented populations in research and healthcare; and (4) Research teams need training in cultural competence and humility. These themes highlight barriers to research, illustrate research priorities and concerns, and provide methods to enhance engagement in research from the perspective of underrepresented populations (See Table 1 of how these themes compared across groups.)

**Table 1:** Themes indicated across community listening sessions

Group N = 117	Themes				Number of themes within group
	Uncertainties of underrepresented populations regarding research participation	Ineffective communication about research opportunities and research findings	Research on primary care and prevention are priorities for underrepresented populations in research	Research teams need training in communication and cultural humility	
African American Men n = 7	X	X	X		3
Deaf and hard of hearing n = 10		X	X	X	3
Low socio-economic status African Americans n = 11	X	X	X		3
Low-income African American parents n = 12	X	X	X		3
Low socio-economic status Caucasians n = 7	X	X	X	X	4
Uninsured Caucasians/African Americans n = 9	X	X	X		3
Low socio-economic status college students n = 9	X	X	X		3
Older Spanish speaking Hispanic Americans n = 16		X	X	X	3
Low socio-economic status Caucasians/African Americans n = 13	X	X	X		3
Low socio-economic status Caucasians/African Americans n = 11	X	X	X	X	4
Residents of HUD-housing (low-income housing) n = 12	X	X	X	X	4
Number of themes across groups	9	11	11	5	

### Uncertainties of Underrepresented Populations Regarding Research Participation

Community members expressed their insecurities as it relates to their research participation. Lack of trust was commonly cited among community members. In addition to lack of trust, community members expressed their concerns for safety and how it influences their participation or lack thereof in research. Each of these will be discussed in further detail below.

#### Lack of Trust

Lack of trust stemmed from past personal and familial experiences with medical research. These experiences resulted in community members perceiving both negative physical and emotional implications towards research. For members who had previously participated in research, there was a sense that researchers were not forthcoming about their research purposes, and in many cases, refrained from providing evidence of progress throughout the research study. Community members also reported a lack of knowledge regarding medications taken or administered as a part of research procedures. Failure to be provided with sufficient explanations of the medications’ purpose and side-effects was commonly reported by community members.

#### Concerns for Safety

In addition to expressing lack of trust, community members conveyed concerns for safety. Concerns about safety were often generated from exposure to media outlets such as television and internet stories. The stories, both fictional and non-fictional, contributed to feelings of skepticism toward the true intentions of researchers and research objectives. Examples of these concerns included fear of being treated as “guinea pigs,” being provided with placebo medications, and loss of life resulting from research participation.

### Ineffective Communication About Research Opportunities and Research Findings

Consequences of ineffective communication result in limited reach of research opportunities to underrepresented populations. It also contributes to lack of research dissemination to these groups. The continuum of communication issues include the sources of communication to the methods (i.e., channels) of communication. The details of these issues are explained below.

#### Limited Reach of Research Opportunities to Underrepresented Populations

A key aspect to engaging community members in research is connecting the community to research opportunities. Ineffective communication was cited as a substantial barrier to connecting underrepresented community members to research. A major contributing factor was lack of awareness of research studies and of the researchers who conduct the studies. Community members expressed that research information, such as project outcomes, is not adequately being disseminated throughout the community. In instances where research information reaches the community, it is not easily understood and is not user friendly for some groups. Additionally, those community members who felt their efforts were futile in identifying research opportunities cited that the information on research websites was inadequate, out of date, and/or too complex. Furthermore, they wanted the research outcomes to be circulated throughout the community to assist its members in making informed healthcare decisions.

#### Lack of Research Dissemination to Underrepresented Populations

Community members identified current and preferred methods for communicating research information and opportunities. Current methods of communication included websites, physicians, family, and friends; however, these methods were characterized as ineffective. Preferred methods of communication identified by community members consisted of community meetings, newsletters, and social media (e.g. YouTube, Facebook).

### Research on Primary Care and Prevention are Priorities for Underrepresented Populations in Research

Research priorities among community members involved research on prevention, need for patient-centered care, and patient-centered outcomes in research, and improved patient-provider communication. Participants reflected on personal experiences and perceptions towards research participation to express their priorities. Each of these areas is discussed in further detail below.

#### Research on Prevention

Primary prevention was a theme that deeply resonated within the listening sessions. Particularly, community members provided their perspective on the need for research aimed at preventing chronic diseases. Diseases identified as priorities include heart disease, diabetes, cancer, and Alzheimer’s disease. Concern for these specific illnesses is a result of the experiences and even loss of many family and community members who suffered with these illnesses. There were also suggestions that exercise programs, organic foods, and natural supplements should be incorporated into the research agenda in an effort to prevent chronic disease. The Community further emphasized the need to identify and/or improve current treatments for these diseases to improve health-related quality of life.

#### Need for Patient-Centered Care and Patient-Centered Outcomes in Research

In addition to establishing research priorities, participants indicated that research should focus on patient-centered care. Community members highlighted the need for patient-centeredness to improve the quality of healthcare. Community member discussions further emphasized their desire to be involved in the research process so that their needs and preferences would be part of improving healthcare delivery and outcomes. Lastly, many members emphasized that researchers and providers are not responsive to their medical needs regarding information delivery preferences and treatment options.

#### Improved Patient-Provider Communication

Community members expressed a profound need to improve patient-provider communication. Poor communication in medical settings was a common experience among community members in each CLS. Several participants suggested that their lack of participation in research was linked to ineffective communication with medical providers and/or researchers. Examples included inadequate information, insufficient explanations of blood test results and treatment options, insufficient time with their providers, and methods of follow-up.

### Research Teams Need Training in Cultural Competence and Humility

The need to improve cultural competency among healthcare professionals and clinical research staff was a primary concern of community members. This perspective was evident in community member personal experiences with medical and research staff. Experiences indicating the need for cultural competence include poor treatment by research staff, cognitive bias among researchers, and lack of willingness to use devices supporting communicate with patients with disabilities. For some community members, these experiences were at their initial research encounter, subsequently tarnishing perceptions of research participation (see Table 2 for examples of participants’ quotes for each theme).

**Table 2:** Themes and examples across community listening sessions

Themes	Examples from transcripts
Uncertainties of underrepresented populations regarding research participation	▪ “…I don’t feel like they give it to you straightforward if you want to participate. You want to be involved in it, but there’s a sixth sense from things that you’ve seen in the past with medical research…” ▪ “I just think it’s what people have alluded to… if you are participating in research that involves trying out a drug that they are not 100 % sure of how it is going to affect you, I don’t think I’d want to be that guinea pig” ▪ “I was going to say safety, depending on what type of research it is. If they are testing a new drug, what are the possible side effects or any ramifications from something unknown?”
Ineffective communication about research opportunities and research findings	▪ “I was in the E.R. without any interpreter. There was no interpreter, and they refused to get an interpreter.” ▪ “Yeah, I would like to see us get more information in the black community. We don’t get information, pertinent, information. I’m not being racist, but white folks know about what’s going on…” ▪ “You want to be updated on their progress… on what they had learned. They need to show you proof that they are making progress…”
Research on primary care and prevention are priorities for underrepresented populations in research	▪ “I would like to see more focus on preventative healthcare … you know, the things that we should do to prevent these things from happening, to prevent heart disease, diabetes and stroke.” ▪ “…one time I went to do the research. I had never taken anything. One set of nurses were so condescending…Her energy was not good. I wasn’t going to come back. It was just horrible.” ▪ “…most of the time the doctors will say, “You’re okay.” Okay, then what are my numbers? What are my levels? What are my blood sugar levels? They don’t say anything. They just say, “You’re okay.” So, really, I want to find out what’s going on”
Research teams need training in communication and cultural humility	▪ “It makes me wonder… do you think there’s a stigma attached to the people, for example, who get the food stamps? Maybe that’s why we don’t talk about it as a society…” ▪ “Sometimes when I tell them I’m deaf, they give me a piece of braille paper.” ▪ “I participated in research and I done it about three times… One was about body fat. They said I was obese. How dare they?”

## Discussion

Eliminating disparities in health and health care is a top priority identified by the US Department of Health and Human Services’ Healthy People 2020 initiative [31]. Increasing the participation of underrepresented populations in research is a mechanism to reduce health disparities [3]. Though there is a long-standing mandate, *the National Institute of Health Revitalization Act*, for the inclusion of women and minorities in research [9], few studies have made it a priority to incorporate underrepresented populations throughout the research process [26]. This study illustrates the mutual benefit/shared authority of an academic-community partnership to capture health and research priorities along with research experiences among a diverse group of underrepresented populations.

The NRC played a key role in the design and implementation of the sessions by recruiting participants and creating, reviewing, and providing feedback on the materials used during the sessions. The NRC helped set the geographical boundaries for the listening sessions based on their experience and relationship with the population of interest. Our target audience, identified by community leaders, are often overlooked within the research enterprise: HUD housing communities, Spanish-speaking communities, and individuals with disabilities. The conversational nature of the CLSs and engagement with the community during the process enabled us to identify and prioritize the health and research concerns of underrepresented communities surrounding the Nashville surrounding area.

Defining research priorities is essential to guiding science, research development, and improving health outcomes. Top health and research priorities from the community perspective were identified; a novel contribution to the literature. Since community members rarely have the opportunity to provide input in health research, it was important to gauge their areas of priority. Priorities included cancer, Alzheimer’s disease, cardiovascular disease, and diabetes. Perceived barriers to research, both physical and behavioral, were consistent with the literature [17, 21]. Participants with positive research experiences reported more positive views towards research than those who had limited knowledge of research, or those knowledgeable of research and its historical abuses [32]. Participants further stressed the need to gain more knowledge on research in general, as well as mechanisms to provide information on researchers and studies. Compelling suggestions were to receive information through community meetings (implying researchers must be visible in the community), educating the community on the “nature” of research, and being responsive to community-preferred modes of research dissemination. Last, this study demonstrates that while providers play a major role in their patients’ health and research decision-making, they engage in ineffective communication on the topics of concern to the CLSs. Identifying mechanisms that promote patient-centered medical homes and research opportunities are necessary to improving individuals’ comfort in engaging in research and preventive health activities.

### Lessons Learned

Similar to past studies on barriers to minority participation in research [33], we found that these underrepresented populations had queries concerning their potential participation. Example questions from these participants included: (1) “Where and how do I find out about research studies?” (2) “What is the purpose of participating in research?” (3) “How will this study affect my safety?” and (4) “What happens when the study is complete?” As an initial step to address these concerns, we developed a frequently asked questions handout that we shared with the participants at the completion of the sessions. This handout explained the benefits of participating in research, legal safeguards that are in place (IRB, informed consent, and the Patient Bill of Rights), and local resources to learn about opportunities to participate in research. To further address these concerns, Table 3 provides recommendations informed by study participants for engaging underrepresented populations, ideas that can potentially keep them engaged in research.

**Table 3:** Recommendations for engaging underrepresented populations

Establish a line of communication between researcher and community during all phases of research
Broadly disseminate research opportunities implementing user-friendly strategies
Transparency regarding risks of research to participants and community
Distribution of more comprehensive, up-to-date information on clinical research and researchers
Recognition of community members as partners in research
Build trust between community, academicians, and clinicians by teaching these individuals to engage in effective, bi-directional communication among these groups. This will help to gain an understanding of each stakeholders research needs in order to improve research participation of underrepresented groups
Use of engagement strategies to ensure communications are person-centered

### Strengths

The community was engaged in all phases of the research process, including the development of this paper, a process ensuring the community voice heard. Because this study was exploratory and descriptive in nature, the researchers gained insight on local health and research experiences (e.g., barriers, concerns), as well as underrepresented populations’ expectations in order to participate in research. An in-depth understanding was gained through the participant-driven CLSs, the free-form approach which provided content-rich themes, and the use of the constant comparison method [16].

### Limitations

The results of this study may not be generalizable to other underrepresented populations beyond the Nashville, Davidson county corridor of Tennessee. However, we purport the findings will provide valuable guidance for conducting CLSs in other similar areas and with other underrepresented populations. Lastly, it is plausible that researchers’ personal biases could interfere with the collection of data and interpretation of the results; however, the CLSs were facilitated by the community partner and the transcriptions were provided verbatim for analysis, minimizing researchers’ influence on the data. The subsequent theme analyses were double coded and divergent codes resolved by consensus.

### Public Health Implications

This study sought to implement an innovative, community-engaged research approach to identify research priorities of underrepresented populations while documenting the concerns that contribute to poor rates of participation in research. Results of this study and more like it could be leveraged to impact health disparities that are due in part to poor community engagement. Further studies are needed to determine if these findings resonate with hard to reach populations in other geographical areas. Application of this approach to highlight health priorities, research experiences, and the research needs of these populations could potentially unmask new strategies to facilitate underrepresented community members’ involvement along the entire research continuum.
